# Phoswich Detectors in Sensing Applications

**DOI:** 10.3390/s21124047

**Published:** 2021-06-11

**Authors:** Sujung Min, Bumkyung Seo, Changhyun Roh, Sangbum Hong, JaeHak Cheong

**Affiliations:** 1 Department of Nuclear Engineering, Kyung-Hee University, Yongin-si 17104, Korea; sjmin@kaeri.re.kr; 2 Decommissioning Technology Research Division, Korea Atomic Energy Research Institute, Daejeon 34057, Korea; bumja@kaeri.re.kr; 3 Quantum Energy Chemical Engineering, University of Science and Technology (UST), 217 Gajeong-ro, Daejeon 34113, Korea

**Keywords:** sensing application, phoswich, radiation, detection, measurement, scintillation materials

## Abstract

Herein, we review studies of the integration of Phoswich detectors with readout integrated circuits and the associated performance in a radiological sensing application. The basic concept and knowledge of interactions with scintillation materials and the mechanisms and characteristics of radiological detection are extensively discussed. Additionally, we summarize integrated multiple detection systems and Phoswich detectors in radiological measurements for their device performance. Moreover, we further exhibit recent progress and perspective in the future of Phoswich-based radiological detection and measurement. Finally, we provide perspectives to evaluate the detector performance for radiological detection and measurement. We expect this review can pave the way to understanding the recent status and future challenges for Phoswich detectors for radiological detection and measurement.

## 1. Introduction

Radiological detection and measurement for accurate radioactivity is an essential process in many areas in nuclear facilities, including decommissioning sites, nuclear security, nuclear safeguards, nonproliferation, and radiation science and technology. In general, a detection system (a scintillation detector, a semiconductor, etc.) is selected in consideration of various factors such as volume, material, density, etc. of the object to be measured, as well as the type, efficiency, and MDA of the radiation measurement system. Recently, research has been conducted to minimize the effect on the background, which is the energy scattered by interacting with target materials, environmental radiation, and cosmic rays. The MDA (minimum detectable activity) is a level of activity concentration that is practically achievable by an overall measurement method, while the detection limits only provide information about the intrinsic performance of the instruments. Typically, since the MDA value increases due to the influence of Compton scattering during the interaction between a photon and material, this effect should be minimized to increase the reliability of the analysis value for the energy of the region of interest [1,2,3]. The MDA includes several factors that affect not only background and detection efficiency, but also sample size, measurement time, and self-absorption. The MDA varies depending on not only the characteristics of the measuring instrument, but also the characteristics of the sample, measurement environment, and method. Therefore, when the analysis method is improved or the measurement equipment is replaced, the MDA should be recalculated. If the measurement time is extended indefinitely for a low MDA, the background is also increased, so there is a limit. Therefore, research to lower the MDA is needed for accurate radioactivity analysis. The Compton suppression system being studied worldwide is a system for lowering the MDA by reducing the influence of Compton scattering, and it can more clearly perform various gamma peak analyses in the Compton continuous region. Currently, the Compton suppression system mainly uses a main detector composed of HPGe with a good resolution and a guard detector composed of an inorganic scintillator (or plastic scintillator). In addition to the Compton suppression system, an anti-coincidence method is used to remove signals generated simultaneously from the guard detector and the main detector. There are several types of radiation detectors. GM (Geiger–Müller) counters that measure radiation in the air, proportional counters, ion chambers, scintillation detectors, and semiconductor detectors are used. However, for precise measurements in the field, a scintillation detector and a semiconductor detector are used [2,3,4,5,6].

**Figure 1:**
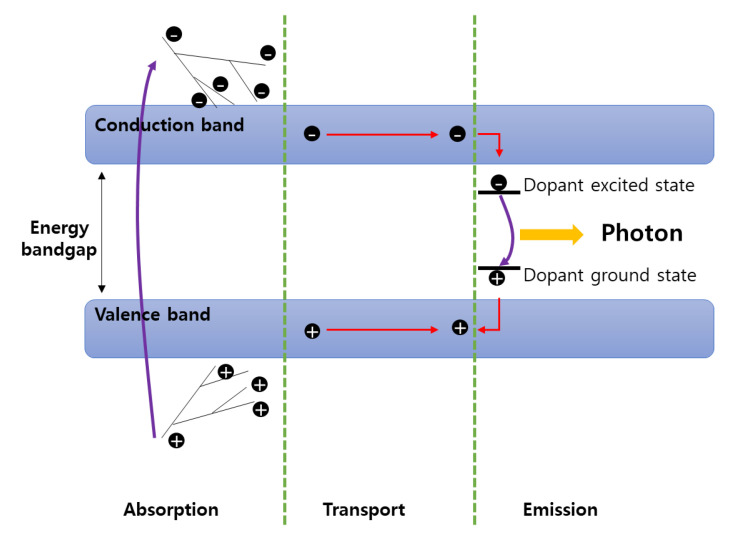
Schematic illustration of the various stages involved in the scintillation process modified [14,15,16].

The scintillation detector is a detector made of a scintillation material that converts absorbed radiation energy into light in the visible region. As shown in Figure 1, some scintillation material in the scintillator is excited by incident radiation. Due to excitation by ionizing radiation, several pairs of hot energetic electrons and holes are generated. These electrons and holes are called carriers, and the recombination of carriers occurs after the transport step in which the carriers can be trapped at the defect level. During recombination, light is generated, and the generated light is amplified by an optical sensor, and an electric signal is generated. The energy and wavelength of the emitted photons are related to the gap energy level of the dopant. The best condition as a scintillation material is that the efficiency of converting incident energy into light must be good (large light yield), and the transition of electrons must be fast (short decay time), and the refractive index of the material should be similar to glass in order to lower the probability of reflection of light. There are two commonly used types of scintillators: inorganic and organic. The scintillation mechanism is different for these two types. Inorganic scintillators typically include NaI(Tl), CsI(Tl), BGO, and LaBr_3_(Ce), and organic scintillators include organic crystals, organic liquids, and plastic scintillators depending on the state of the material. The inorganic scintillator has high gamma-detection efficiency, high luminous efficiency, and a good proportion of the amount of generated scintillation and incident photon energy. However, the inorganic scintillator has a long decay time of scintillation, and NaI (Tl), which is commonly used, is vulnerable to mechanical/thermal shock and has poor processability. In addition, it must be sealed in an aluminum container because it is hygroscopic and sensitive to temperature and humidity. CsI(Tl) has a large absorption of gamma rays per unit length among scintillators. Therefore, there are many fields that can be utilized because the size can be reduced. The BGO (Bi_4_Ge_3_O_12_) detector became commercially available in the late 1970s, and the advantage of BGO is that it consists of a high density (7.13 g/cm^3^) and a large atomic number (83), such as bismuth (Bi). However, it was confirmed that BGO had a relatively low light yield and a light yield of 10–20% NaI (Tl). In addition to the inorganic scintillator described in Table 1, there are ZnS (Ag), YAlO_3_ (YAP), Y_3_Al_5_O_12_ (YAG), LiI (Eu), etc. Table 1 shows the types and characteristics of the inorganic scintillator. The organic scintillator has the advantage of short decay time, strong mechanical/thermal impact, and good processability. However, since the organic scintillator is composed of a material with a low atomic number, it is not mainly used for gamma-ray measurement, and has a disadvantage in that the luminous efficiency of the scintillator can be easily deteriorated due to the influence of the solvent type or impurities [1,2,3,4,5,6,7,8,9,10,11,12]. In addition to the intrinsic inorganic scintillator using halides, studies on new scintillators such as garnets and perovskites have recently been conducted. Perovskite is a general term for ABX_3_ materials and has a three-dimensional crystal structure made by combining two types of cations and one type of anion. Perovskite shows excellent properties for the detection of direct-ionizing radiation due to its very good charge carrier mobility [13].

The semiconductor detector generates electrons and holes corresponding to the ion pairs of the gas by ionization, collects them in an electrode, and measures them. The semiconductor detector has a good resolution and good proportionality between the energy of the incident radiation and the signal pulse. However, there is a disadvantage in that it should be cooled with liquid nitrogen when using it, since the current always flows due to thermal excitation at room temperature as well as the energy absorption of radiation.

**Figure 2:**
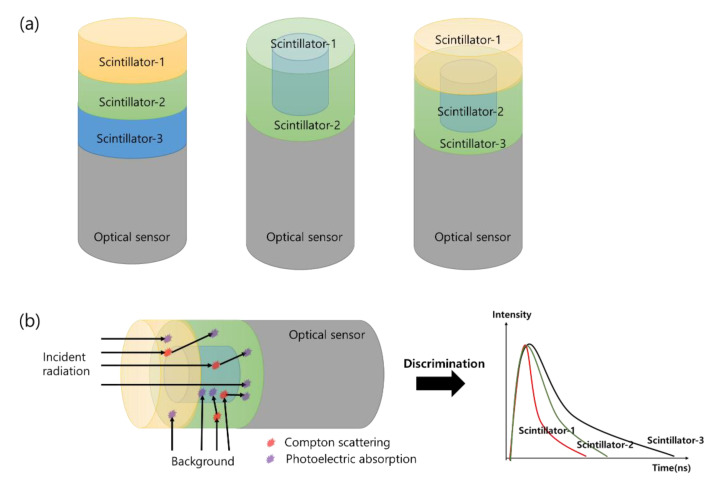
Example of a Phoswich structure (**a**) and example of signal classification method (**b**).

Several types of detectors are composed of single or multiple modules and are used as a radiation-measurement system. If the measurement location is narrow, the scintillator is used in a Phoswich structure for the miniaturization and simplification of the system. Originally, the Phoswich detector was used to measure low-energy X-rays in a gamma-ray background environment. A Phoswich detector consists of two or more scintillators and one optical sensor (PMT or SiPM, etc.). After receiving signals from different scintillators in one PMT, the signals are separated through a data-processing algorithm. Figure 2a is an example of a Phoswich structure, and Figure 2b shows an example of discriminating signals from different scintillators. The Phoswich structure can be configured differently depending on the user. Since the decay time is different for each scintillator, the signal can be discriminated as shown in Figure 2b. 

The influence of external radiation and the effect of Compton can be eliminated. However, since multiple signals are received by one optical sensor, signal crosstalk may occur. In other words, signals received from one optical sensor can cause unintended effects on other channels. Therefore, many studies are being conducted to reinforce the advantages of the Phoswich detector and solve the problems. Phoswich detectors can be miniaturized, and data accuracy can be improved because only signals in the region of interest are collected by removing unnecessary signals. In addition, since it is possible to discriminate signals using different decay times for each scintillator, it is possible to simultaneously measure various radiations. 

In this review, we summarized integrated multiple detection systems and Phoswich detectors in their radiological measurements for their respective device performances. Additionally, we investigated the integration of Phoswich detectors with readout integrated circuits and the associated performance to bridge the gap of shortcomings of the existing background suppression system, the current state of technology for the composition, signal processing method, and application method of the Phoswich detectors.

## 2. Radiation Measurement Technologies

### 2.1. Beta Nuclides Measurement Technologies

H-3 and C-14 are representative nuclides that emit beta rays, and currently, many studies are being conducted to measure H-3 in water as well as air. H-3 is a radioactive material that is produced during nuclear tests, but also during nuclear reactor operation and nuclear fuel reprocessing. H-3 has a half-life of 12.35 years and emits beta rays of up to 18.54 keV and an average of 5.69 keV. Beta rays generated from H-3 have weak energy and very low permeability, so the path length (range) in the air is about 6 mm, and the range in water is about 5 μm. Accordingly, there is a need for research on the development of a new radiation sensor for detecting nuclides-emitting beta rays [2,26,27,28].

In 2002, Milan-INFN University from Italy developed a system using a plastic scintillator for beta-ray measurement. The scintillator used in this study was a plastic scintillator (NE102A) with a 76.3 mm (3-inch) diameter and 50 mm thickness purchased from Nuclear Enterprises. The point of this study is a rectangular well of 3 cm × 2 cm × 1 cm size in the center of the plastic scintillator, and a measurement sample placed in it. The device is a measurable system in a laboratory environment, and high-energy and low-energy calibrations were performed using Sr-90 and Am-241 sources, respectively. In addition, verification of the device was performed through MCNP modeling, and performance was verified by comparing and analyzing the computational simulation results and experimental values based on the beta-ray spectra of H-3 and C-14. 

**Figure 3:**
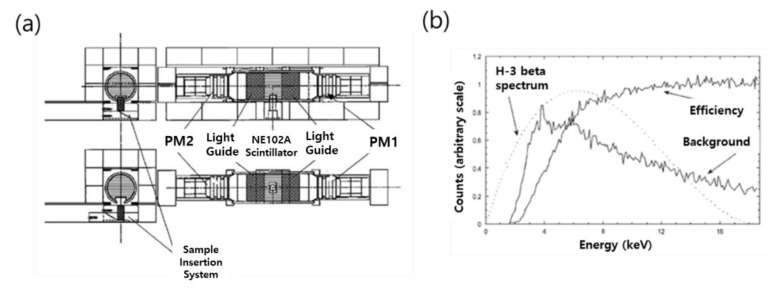
Drawing of a plastic detector for beta-ray measurement (**a**) and energy spectrum (**b**) [29].

As shown in Figure 3b, the sensitivity calculation takes into account the interval (6 keV~14 keV) between the energy at which the H-3 spectrum reaches its maximum value and the background falls slower than the beta spectrum. The measurement time was 1 day, and as a result of calculating the MDA for the 90% confidence level, it was calculated as 1.21 Bq/cm^2^. The MDA was calculated through the equation below [29]:

(1) $$\mathrm{MDA}=\frac{\left(\frac{k^{2}}{T_{S}}\right)+2k\sqrt{(\left(\frac{R_{B}}{T_{B}}\right)+\left(\frac{R_{B}}{T_{s}}\right)}}{E\left(\frac{A}{100cm^{2}}\right)}$$

where *R_B_* is the count rate of the natural background, *T_B_* is the time of the background count, *T_S_* is the time of the sample count, *E* is the detection efficiency, *A* is the area monitored (cm^2^), and *k* is the number of standard deviations (*k* = 1.65 corresponding to a confidence level of 90%).

**Figure 4:**
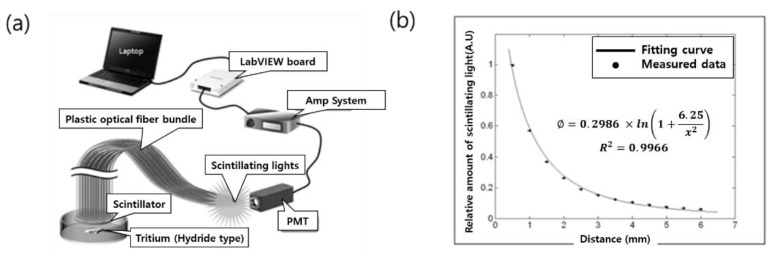
Configuration of the optical fiber-based sensor for detecting tritium (**a**) and the relative amount of scintillation (**b**) [30].

In 2009, Konkuk University from South Korea manufactured an optical fiber radiation sensor for detecting H-3 using an inorganic scintillator and optical fiber. For the selection of inorganic scintillators, sensor tips of Gd_2_O_2_S:Tb, Y_3_Al_5_O_12_:Ce, and CsI:Tl were manufactured, and then the scintillator with the best scintillation efficiency was selected using H-4 in the form of metal hydride [30]. In addition, the amount of scintillation according to the distance from the tritium source was measured using the manufactured sensor, and the amount of scintillation according to the intensity of the radioactivity of the source was measured and compared with the result of surface radioactivity. Figure 4a shows the overall experimental configuration, and the scintillation light generated from the scintillator by metal hydride H-3 is transmitted to the PMT by a 1 m long optical fiber, converted into an electric signal, and amplified by an amplifier system.

As a result of the experiment, the amount of scintillation of the Gd_2_O_2_S:Tb sensor was the largest and reacted most sensitively to the source. As a result of measuring the amount of scintillation according to the distance from the tritium source with the Gd_2_O_2_S:Tb sensor, the amount of scintillation decreased as the distance from the source increased, as shown in Figure 4b. The dose at x distance from the disk radiation source is determined by the equation below. The amount of scintillation for H-3 with a path length (range) of 6 mm was about 6% when the distance was 0.5 mm.

(2) $$\Phi =\frac{S}{4}\times \ln\left(1+\frac{r^{2}}{x^{2}}\right)$$

Here, *S* is a constant representing the strength of the source, and *r* is the radius assuming a disk-shaped source [30].

**Figure 5:**
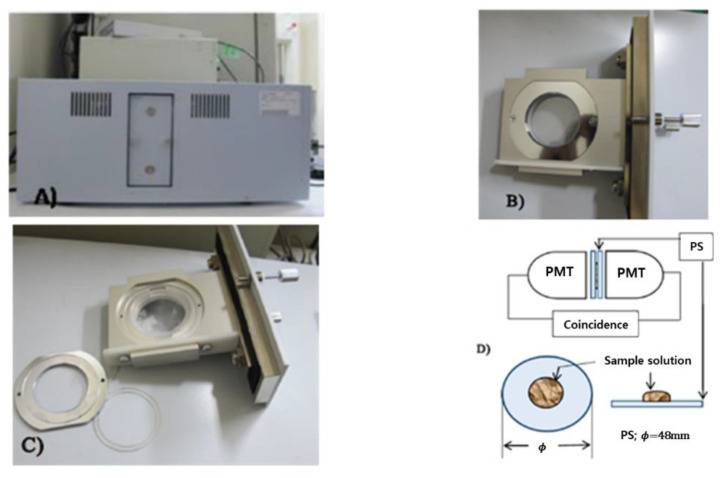
A system designed to measure low-energy beta rays [31].

In 2015, Ochanomizu University from Japan conducted a study on the development of a detector for beta-ray(H-3) spectroscopy, which is low energy, using a plastic scintillator [31]. Figure 5 is a system diagram for measuring a low-energy beta sample. Two circular plastic scintillator plates faced each other, and a source entered between the scintillators. The noise of PMT was removed using a simultaneous summation circuit on two plastic scintillators, and the linearity between the radioactivity and count values of ^3^H-methionine, ^14^C-arginine, and ^35^S-methionine sources was analyzed. As a result of measuring with the system manufactured, a minimum detection limit of 0.0116 Bq/mL was obtained when measuring 2 mL of sample for 10 h. However, this system has the disadvantage that the amount of measurable samples is very small.

**Figure 6:**
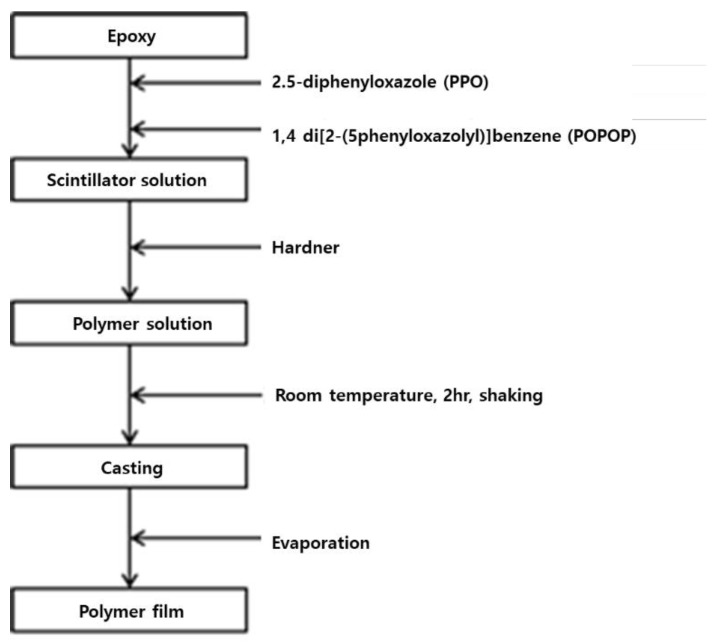
Plastic scintillator manufacturing procedure [32].

In 2017, The Korea Atomic Energy Research Institute produced an epoxy-based plastic scintillator for beta measurement and optimized the amount of added fluorescent material. Plastic scintillators were produced by polymerization after adding a fluorescent substance to a benzene-group solvent. The manufacturing process of plastic is shown in Figure 6. In this study, plastic scintillators were manufactured by 2 mm, 4 mm, 6 mm, 8 mm, and 10 mm thickness, as well as performance comparisons by fabricating them while varying the contents of PPO and POPOP. The radiation source was measured by attaching an Sr-90 point source to the plastic surface. The point of this study is that the optimum thickness (4 mm) of the large-size plastic scintillator for beta measurement was derived, and the optimum manufacturing conditions (polymer: PPO: POPOP = 0.79 wt%: 0.2 wt%: 0.01 wt%) were derived [32,33].

In 2018, Myongji University from the Republic of Korea conducted a study on the detection efficiency of each optical sensor (PMT, SiPM) for the CaF_2_(Eu) scintillator in order to conduct the underwater Sr-90 detection study. SiPM-CaF_2_(Eu) and PMT-CaF_2_(Eu) detectors were prepared, respectively, and Sr-90 contaminated samples at a concentration of 165–84,400 Bq/L were evaluated, and the detection limits (MDA) of the two detectors were calculated. In the case of the SiPM-CaF_2_(Eu) detector, the MDA was calculated as 1319 Bq/L, and the MDA of the PMT-CaF_2_(Eu) detector was calculated as 330 Bq/L. Since the PMT-CaF_2_(Eu) detector has about a four-fold lower MDA than the SiPM-CaF_2_(Eu) detector, the PMT-CaF_2_(Eu) detector is more efficient when detecting low concentrations of Sr-90 contamination [34,35]. In 2019, Lancaster University from the UK demonstrated a study for the detection of tritium in water. In the study, a CaF_2_:Eu scintillator, an inorganic scintillator, was fabricated with a diameter of 28.6 mm and a thickness of 1 mm, a tritium detection device was constructed, and a characteristic evaluation was carried out.

**Figure 7:**
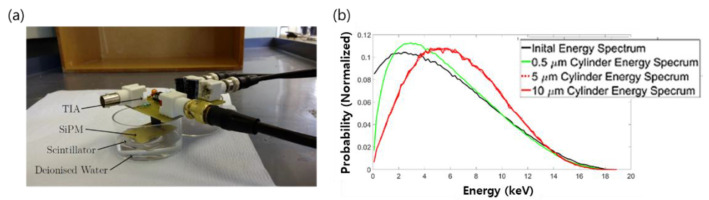
Tritium detection system using the CaF_2_:Eu scintillator and the Preamp(TIA), SiPM (**a**) and energy spectrum according to tritium cylinder thickness (**b**) [28].

As shown in Figure 7a, a CaF_2_:Eu scintillator was fabricated by connecting SiPM operated at a lower voltage (29.7 V) than PMT (1~2 kV). Using this system, the test was performed with a sample of 1500 Bq/mL of tritium prepared in a 20 mL cylinder. The experiment was conducted with different factors to maximize the energy that the beta particles reach in the CaF_2_:Eu scintillator. In addition, the experimental value and the simulation value were compared using Geant 4. The main point of this study was to observe the self-attenuation effect of tritium through the shift of the beta spectrum. As experiment results confirmed, the energy spectrum shifted according to the thickness of the tritium cylinder as shown in Figure 7b, and it was confirmed that the specific crystallinity of the beta particles in water was 0.3 um or less. The reason for the shifting of the energy spectrum is due to the self-attenuation effect of the source, and it was analyzed that the self-attenuation effect of tritium was about 5 μm from the surface of the scintillator, as the 5 μm and 10 μm thickness showed the same energy spectrum shape. In addition, in this study, when preparing a CaF_2_:Eu scintillator, it was confirmed that the smaller the particle size, the higher the counting rate. It was analyzed that the counting rate increased because the cross-sectional reaction area increased when the particle size was small [28].

### 2.2. Gamma Nuclides Measurement Technologies

In general, a scintillation detector for gamma-ray detection should have good luminous efficiency, good light transmittance, and short decay time when radiation is incident. In addition, the spectral sensitivity with the optical sensor should be suitable, and it should be similar to the refractive index of the glass in order to effectively contact the optical sensor. Since the gamma-ray measurement is easier to analyze than alpha and beta, many studies have recently been conducted to develop gamma nuclide measurement technology for site and water and to secure a low MDA for a gamma measurement system. 

Therefore, in this section, research cases on minimizing background effects for securing a low MDA are investigated and described. In general, HPGe with good energy resolution is mainly used when analyzing nuclides, but HPGe has a disadvantage in that it has a size limitation and requires cooling during measurement. In addition, since the measurement time cannot be infinitely increased, research on lowering the MDA by reducing the background is being conducted globally.

**Figure 8:**
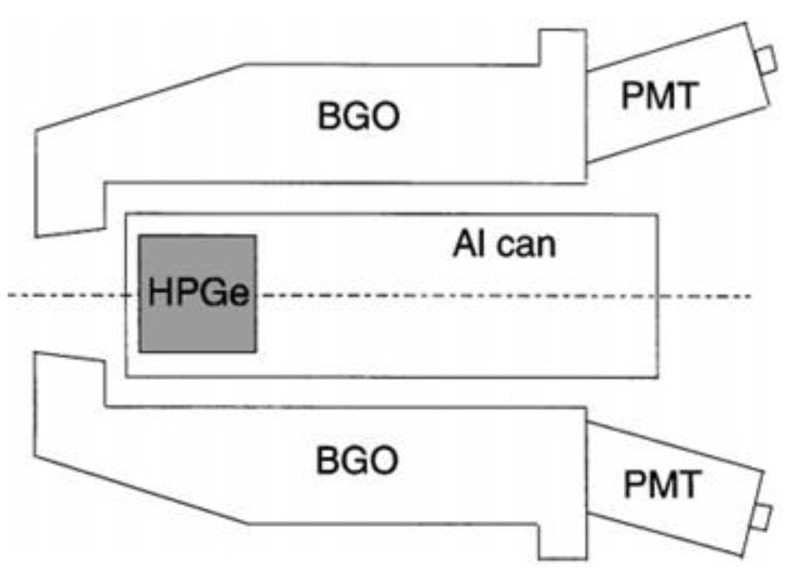
Compton suppression system manufactured by California University [36].

**Figure 9:**
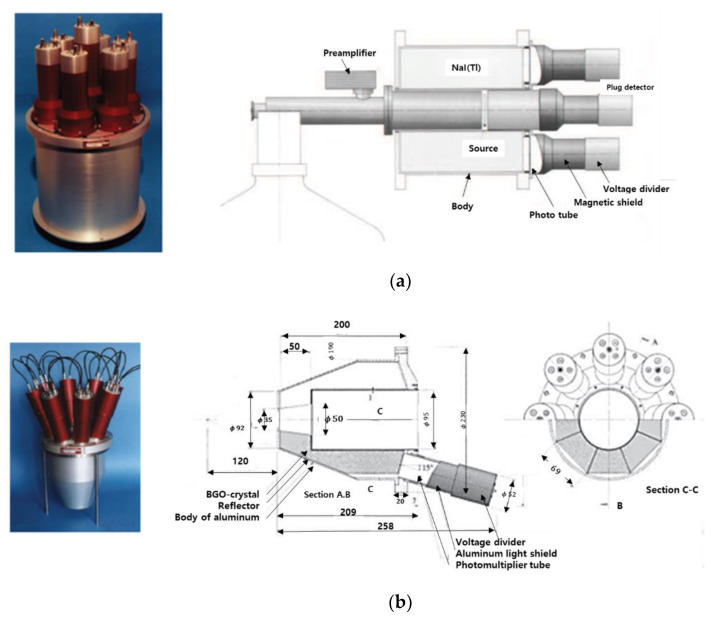
Compton suppression system using HPGe and a guard detector. (**a**) NaI(Tl) is used as a guard detector (**b**) BGO is used as a guard detector [37].

**Figure 10:**
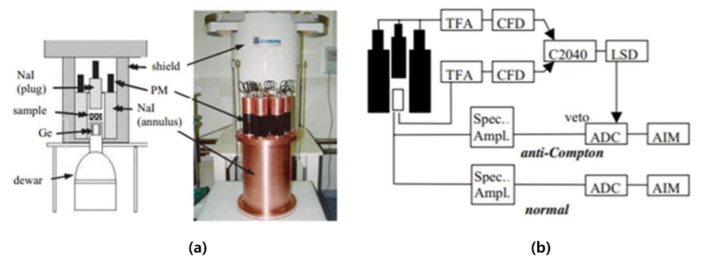
Anti-Compton system(**a**) and circuit diagram(**b**) [38].

In 1999, the University of California from the US conducted a study on Compton suppression of HPGe using the Pulse Shape Analysis (PSA) method [36]. The advantage of the PSA method is that it can eliminate not only reactions that escaped from a single region, but also reactions that escaped from multiple regions. In this study, as shown in Figure 8, a Compton suppression system was fabricated and tested using HPGe as the main detector and BGO as the guard detector. It is a structure in which BGO surrounds a 5 cm × 5 cm sized HPGe crystal. As a result of experiments using Eu-152 nuclides, it was confirmed that efficiency increased by 30% through the removal of Compton at 964 keV. In addition, Berkeley Nucleonics Corp (BNC) of the United States sells many types of detectors, among which the Compton suppression system using HPGe has also been commercialized and sold. Gamma rays generated by Compton scattering create a continuous background in the gamma-ray spectrum, resulting in a higher MDA. Therefore, the effect on Compton scattering should be eliminated. Figure 9 shows the Compton suppression system sold by BNC [37]. Figure 9a has a structure that can detect gamma rays scattered from the front. However, this product has a limited source size. Figure 10b is similar to the above-mentioned research results at the University of California and has a structure in which HPGe is surrounded by BGO, not NaI(Tl). The decisive parameters of the anti-Compton system are the angle of the guard detector and the high stopping power. BNC generally recommends the product of Figure 9b type, except for systems that require a low background. When low background radiation is required, a product using NaI(Tl) as a guard detector is recommended. NaI(Tl) has a 500% higher light output than BGO, and NaI(Tl) has many advantages because it can be manufactured in a larger size than BGO.

**Figure 11:**
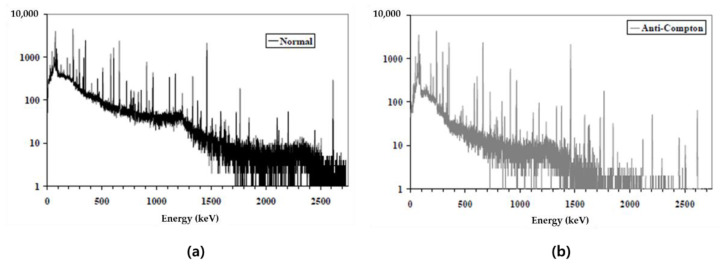
Compton suppression results using U, Th source (**a**) Spectrum with Compton suppression unapplied (**a**) and applied spectrum (**b**) [38].

In 2009, IRSN from France conducted a study on minimizing the influence of Compton scattering using the anti-coincidence method, and an anti-Compton system as shown in Figure 10 was developed [38]. The anti-coincidence method is called anti-coincidence counting, and it is the principle of counting after removing the signal from the MCA instead of recording it when the signal comes in at the same time. The signal-processing circuit is composed of each Spectroscopic amp, ADC, and AIM. As shown in Figure 11, the performance of removing the effects of Compton can be confirmed. In particular, the performance of removing the effects of Compton by natural radionuclides, the daughter nuclides (1764 keV, 2614 keV) of U-238 and Th-232, was confirmed. RF (Reduction Factor), which is a removal performance index, is defined by the following equation:

(3) $$RF=\frac{n_{N}}{n_{AC}}$$

Here, *n_N_* is a counted value (general spectrum) without an anti-Compton system, and *n_AC_* is a counted value (Compton suppression spectrum) when an anti-Compton system is used. As a result of calculating the MDA using the Compton suppression system, the MDA of Co-60 decreased from 8.2 ± 0.4 Bq/kg to 1.8 ± 0.2 Bq/kg.

**Figure 12:**
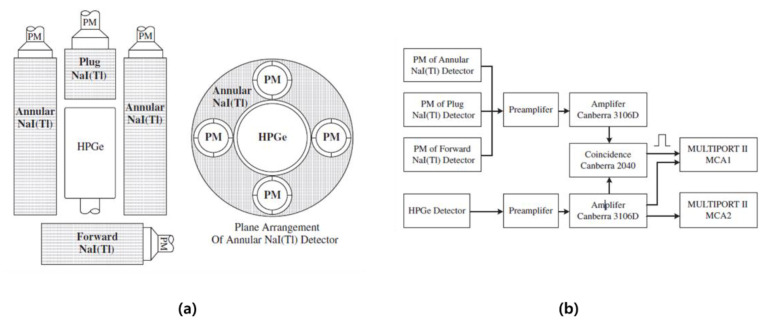
Compton suppression system (**a**) and electronic circuit (**b**) fabricated by CTBT Beijing National Data Centre [39].

In 2013, CTBT Beijing National Data Center from China demonstrated a study to improve the performance of the Compton suppression system composed of a HPGe detector and three NaI(Tl) detectors [39]. In this study, aerosol samples collected in Beijing after the Fukushima nuclear accident in Japan were used, and Compton suppression and non-suppression spectra were analyzed. The Compton suppression system manufactured in this study is shown in Figure 12a, and the electronic circuit is shown in Figure 12b. As a method of removing the signal when the NaI (Tl) signal and the HPGe signal are detected at the same time, the gate is controlled in MCA 1 and the Compton suppression spectrum is stored in MCA 1. Additionally, the Compton non-suppression spectrum obtained by HPGe is stored in MCA2. Three samples with different collection times were prepared, and as a result of performing Compton suppression and non-suppression tests, the peak of the Compton ratio in the energy region of cesium nuclides was 0.26 (604.72 keV), 0.20 (795.86 keV), and 0.06 (569.33 keV). In addition, the MDA of Cs-137 was calculated as 1.5 μBq/m^3^ (Compton non-suppression) and 0.7 μBq/m^3^ (Compton suppression), respectively. It was confirmed that the MDA was lowered through the Compton suppression system.

**Figure 13:**
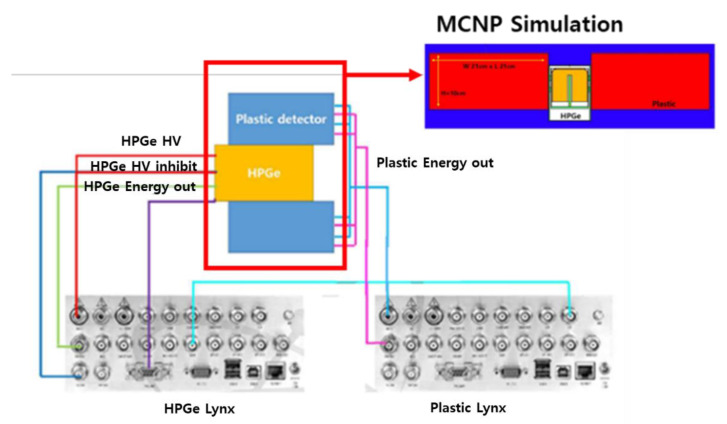
Configuration diagram of Compton suppression system circuit processing device [40].

In 2015, the Korea Atomic Energy Research Institute conducted research on background reduction using HPGe as the main detector and a plastic detector as the guard detector. The performance was evaluated by comparing the ratio of the Compton continuous region and the photoelectric peak region when the background reduction method was applied (suppressed) and the background reduction method was not applied (unsuppressed). According to a 2015 study [40] in a system configured as shown in Figure 13, when gamma rays emitted from the source enter the plastic guard detector and then the generated scattered gamma rays enter the HPGe main detector, this signal is counted in the Compton continuous region. Therefore, as shown in Figure 13, a plastic guard detector is placed around the back of the HPGe main detector, and the gamma rays scattered from the main detector can be detected by the guard detector. At this time, a signal processing device was constructed so that the simultaneously generated signals are not recorded in the main detector signal by using an anti-coincidence [41].

To quantitatively compare the background suppression effect based on the measured gamma spectrum, the results were measured by suppression and un-suppression, respectively, and then evaluated by the following equation:

(4) $$\mathrm{CSF}(\mathrm{Compton}\;\mathrm{Suppression}\;\mathrm{Factor})=\frac{\left(\mathrm{Peak}\;\mathrm{to}\;\mathrm{Compton}\;\mathrm{ratio}\right)\mathrm{unsuppressed}\;}{\left(\mathrm{Peak}\;\mathrm{to}\;\mathrm{Compton}\;\mathrm{ratio}\right)\mathrm{suppressed}}$$

Here, the peak of the Compton ratio is the counts for each energy region and is defined as the ratio of the full energy peak region to the continuous Compton region.

The Korea Atomic Energy Research Institute is continuously conducting research on performance improvement using HPGe as the main detector.

**Figure 14:**
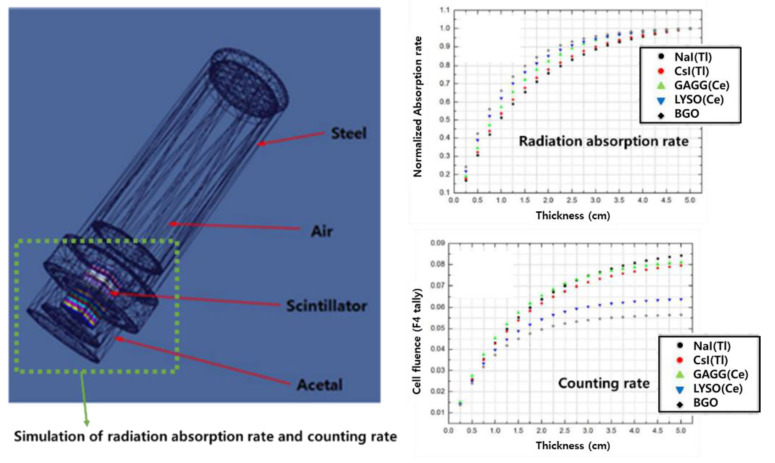
Simulation results of a radiation detector using MCNP6 simulation [35].

In 2019, Myongji University from the Republic of Korea compared the radiation absorption rate and counting rate for each scintillator through the MCNP simulation to develop an underwater gamma-ray detector. Figure 14 shows the geometry of the MCNP 6 computational simulation for the analysis of the radiation absorption rate according to the thickness of the scintillator. The detector housing material was set to acetal to minimize the transmission of gamma rays, and the housing was set to steel for the sensor and circuit parts. The thickness of acetal and steel was 1 cm each. The detection unit was designed in the form of 20 overlapping scintillators of 2.54 cm × 25.4 cm × 0.25 cm. The radiation energy used in the computer simulation was 662 keV, which was the gamma-ray emission energy of Cs-137, and the distance between the detector and the source was 0.1 cm. Figure 14 is a graph of the radiation absorption rate and counting rate according to the thickness of each scintillator. In the absorption graph, it was confirmed that BGO had the highest absorption rate when the scintillator had the same thickness. This is because the interaction with radiation is greatly influenced by the density and effective atomic number. However, as for the generated photons, BGO was the lowest, and it was confirmed that GAGG(Ce), NaI(Tl), and CsI(Tl) showed high photon incidence. Myongji University’s research results are useful in the selection of scintillators for Compton suppression systems using Phoswich detectors [35].

### 2.3. Optical Sensor

The optical sensor is a device that converts light energy into an electrical signal. Since the emission wavelength for each scintillator is different, the selection of an appropriate optical sensor must be made in the research stage. If an optical sensor that is not suitable for the scintillator is used, quantum efficiency, light yield, and detection efficiency decrease. There are PD, APD, SiPM, PMT, etc., with different detection performances in the optical sensor. When selecting an optical sensor, it is recommended to select a sensor suitable for the purpose after investigating the following items [42]:

– Emission wavelength, the intensity of the light;
– Signal to noise ratio (SNR);
– Cost, etc.

The characteristics of PMT, APD, and SiPM were presented in Table 2. PMT, which is commonly used, is a classic optical sensor that finally amplifies an electronic signal about 10^6^ times by passing one photoelectron generated from a photocathode through a multi-stage dynode. It usually operates at a load voltage of 1000 V or higher, and it is large and heavy. Additionally, the operation is limited in a magnetic field.

SiPM (Si APD) works on a similar principle as an Avalanche diode. A very large electric field is formed in the PN junction, and the incident optical signal causes an electronic avalanche (avalanche) process. The signal is amplified with about 10^6^ electrons per photon. In the size of about 1 mm^2^, usually small and independent 1000 diodes operate in Geiger mode, and the response signals of all diodes are combined into one sensor output. This is also called an MPPC (Multi Pixel Photon Counter).

A hybrid photodiode (HPD) is a device that detects incident light by accelerating one photoelectron generated from a photocathode to a high voltage of about 20,000 volts and detecting it using a silicon diode composed of a plurality of pixels under a vacuum tube.

## 3. Multi-Signal Processing Methods

### 3.1. PSD (Pulse Shape Discrimination)

#### 3.1.1. Charge Comparison Method

**Figure 15:**
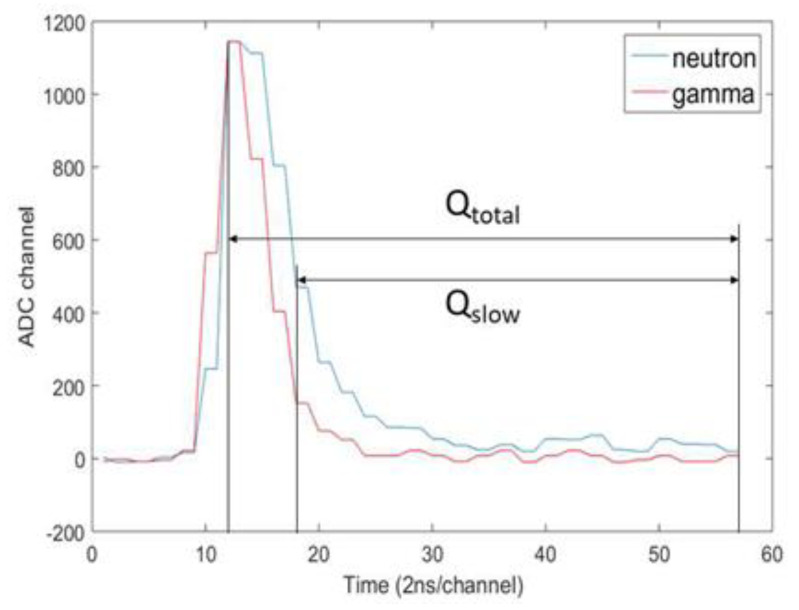
Neutron/gamma-ray signal of the EJ-301 detector with a charge comparison method applied [44].

Since radiation generates different types of signals according to energy, mass, and charge, the type of radiation can be classified using the shape of the signal. The classification of radiation by using the difference in the waveform of the signal generated by the detector is referred to as pulse shape discrimination (PSD). Pulse shape discrimination is basically used to distinguish different radiations (γ, n). Among the PSD methods, the charge comparison method is a method of classifying the type of radiation by using the ratio of the total charge amount of the measured pulse and the charge amount of the falling part. When comparing the PMT output signals of neutrons and gamma rays, the decay time of neutrons is longer than that of gamma rays, so the ratio of the amount of charge in the falling part to the total amount of charge in the neutron is higher than that of gamma rays. The total amount of charge in the pulse is called Q_total_, and the amount of charge in the falling part of the pulse caused by delayed fluorescence is called Q_slow_. The charge comparison method is a method of distinguishing between gamma rays and neutrons by setting Q_slow_/Q_total_ as a PSD parameter. Figure 15 shows the neutron/gamma-ray signal of the EJ-301 detector using the charge comparison method and shows the Q_slow_ and Q_total_ parts [44].

#### 3.1.2. Constant Time Discrimination Method, CTD

**Figure 16:**
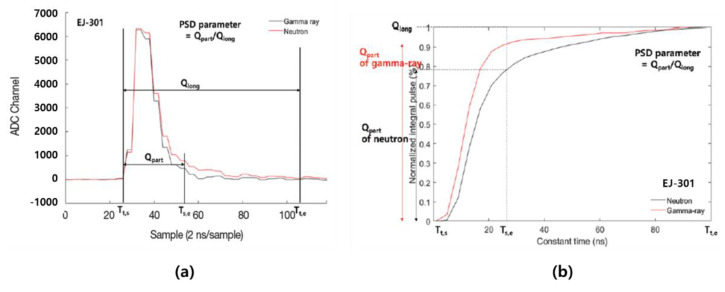
Neutron and gamma signals from the EJ-301 scintillator: (**a**) Signal from digitizer; (**b**) Normalized pulse signal [46].

CTD is a classification method based on the difference in the shape of the integral pulse normalized to the total integral value of the entire signal. As shown in Figure 16, the pulse signal obtained from the digitizer is integrated at 2 ns intervals for a certain period, and the integral pulse is normalized to the maximum value of each signal. Since the decay time of the neutron pulse is longer than that of the gamma-ray pulse, the PSD parameter value of the normalized integral neutron pulse must be smaller than the PSD parameter value of the generalized integral gamma-ray pulse at a certain time.

The PSD parameter of the constant time classification method can be defined as the following equation [45,46]:

(5) $$PSD\;parameter=\frac{Q_{part}}{Q_{long}}=\frac{\int_{T_{t,s}}^{T_{s,e}}Q\;dt}{\int_{T_{t,s}}^{T_{t,e}}Q\;dt}$$

Here, *T_s,e_* is the time after a certain time from *T_t,s_*, and *T_s,e_* is one of the most important elements in CTD.

However, the charge comparison method and the constant time discrimination method (CTD) are not suitable for this Compton suppression system development study, which targets only gamma rays, as a radiation-type classification method that classifies signals of radiation with different characteristics.

### 3.2. FCR–SCR Method

**Figure 17:**
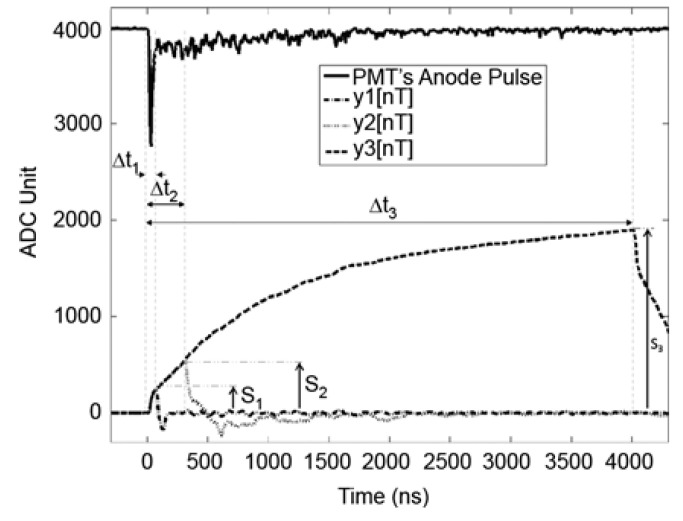
Pulse shape of the Phoswich detector with simultaneous measurement [48].

In order to separate the signal from the Phoswich detector composed of several scintillators, in a study at Oregon State University in the United States [47,48], each anode pulse region was calculated by using a digital triangular filter with different time intervals. Figure 17 is an example of using three digital triangular filters. *y_1_*, *y_2_*, *y_3_* in Figure 17 are the response signal pulses of each filter from the Phoswich detector. *Y[n]* is the sum of the anode pulse signals during the peaking time. Therefore, the amplitude of *y[n]* is the same as the area of the input pulse during the peaking time.

In Figure 17, the output amplitudes of the three filters are defined as *S_1_*, *S_2_*, *S_3_*, and mean the area of the pulse area for each is 60 ns, 300 ns, and 4000 ns after each trigger point, respectively. Using the values of *S_1_*, *S_2_*, and *S_3_*, the calculation of the FCR (Fast Component Ratio) and SCR (Slow Component Ratio) of each pulse can be conducted through the following equation. Since *S_1_* is part of *S_2_* and *S_1_* and *S_2_* are part of *S_3_*, FCR and SCR have a range from 0 to 1. However, in the case of SCR, it was calculated independently from S_1_ (Fast Component) to extract only the tailing part of each pulse. For this reason, the SCR was calculated after subtracting S_1_ from S_2_ and S_3_.

(6) $$FCR=\frac{S_{1}}{S_{2}}\;$$

(7) $$SCR=\frac{\left(S_{2}-S_{1}\right)}{\left(S_{3}-S_{1}\right)}$$

**Figure 18:**
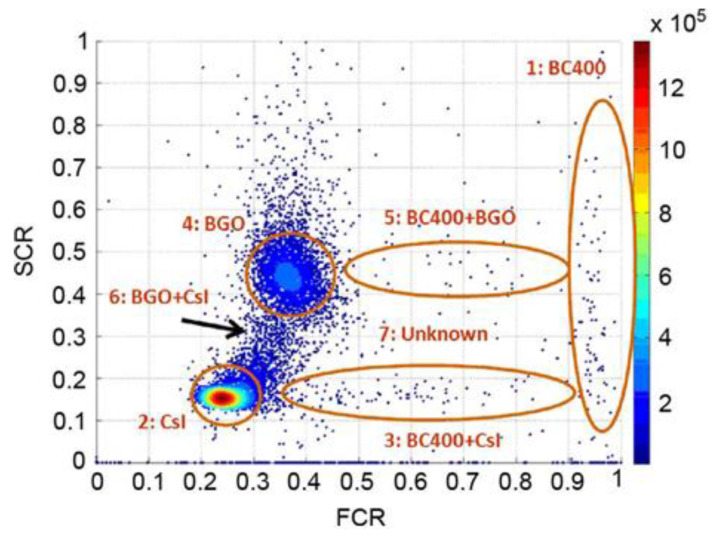
FCR–SCR calculation results using the Cs-137 source [48].

Figure 18 shows the results of testing by the FCR–SCR method using a Cs-137 source after fabricating a Phoswich detector using BC400 (Plastic), BGO, and CsI. Areas 1, 2, and 4 are the single-counting areas of BC400, CsI(Tl), and BGO, respectively, and areas 3 and 5 are the simultaneous measurement areas of CsI(Tl)-BC400 and BGO-BC400. Region 6 is the Compton scattering region between CsI(Tl) and BGO.

### 3.3. Least-Squares Pulse Shape Discrimination Method

The least-squares method is to find a value at which the sum of squared errors between the approximate value and the actual value is minimum. The least-squares method in the PSD method is a method of linearly fitting an experimentally derived function and an actual anode signal. For example, assuming that the Phoswich detector is composed of scintillators BC-400, CsI(Tl), and BGO, the anode signal is calculated by the following equation [47]:

y_i_ = A.*f_A.i_* + B.*f_B,i_* + C.*f_C,i_* +*e_i_* (8)

Here, A, B, and C are unknown coefficients representing the amplitude of the anode pulse generated by the interaction with the three scintillators constituting the Phoswich detector. *f_A_* is the response of the system to the BC-400 scintillator, *f_B_* is the response to the CsI(Tl) scintillator, and *f_C_* is the response to BGO. *e_i_* is the difference between the system’s response and the actual anode signal. The data are fitted by minimizing the sum of squared *e_i_* for the available data, as shown in the equation below.

(9) $$S_{r}=\sum_{i=1}^{n}e_{i}^{2}=\sum_{i=1}^{n}{\left(y_{1}-\left[A\cdot f_{A,i}+B\cdot f_{B,i}+C\cdot f_{C,i}\right]\right)}^{2}$$

To derive A, B, and C in the above equation, they are differentiated by each unknown coefficient (A, B, C).

(10) $$\frac{\partial S_{r}}{\partial A}=2\sum_{i=1}^{n}\left(y_{1}-\left[A\cdot f_{A,i}+B\cdot f_{B,i}+C\cdot f_{C,i}\right]\right)\cdot \;f_{A,i}\;\;$$

(11) $$\frac{\partial S_{r}}{\partial B}=2\sum_{i=1}^{n}\left(y_{1}-\left[A\cdot f_{A,i}+B\cdot f_{B,i}+C\cdot f_{C,i}\right]\right)\cdot \;f_{B,i}$$

(12) $$\frac{\partial S_{r}}{\partial C}=2\sum_{i=1}^{n}\left(y_{1}-\left[A\cdot f_{A,i}+B\cdot f_{B,i}+C\cdot f_{C,i}\right]\right)\cdot \;f_{C,i}$$

If the differentiated value is set to 0, the *S_r_* value becomes the minimum.

(13) $$\sum_{i=1}^{n}\left(y_{i}f_{A,i}\right)=\sum_{i=1}^{n}\left(A\cdot f_{A,i}^{2}+B\cdot f_{B,i}f_{A,i}+C\cdot f_{C,i}f_{A,i}\right)$$

(14) $$\sum_{i=1}^{n}\left(y_{i}f_{B,i}\right)=\sum_{i=1}^{n}\left(A\cdot f_{A,i}f_{B,i}+B\cdot f_{B,i}^{2}+C\cdot f_{C,i}f_{B,i}\right)$$

(15) $$\sum_{i=1}^{n}\left(y_{i}f_{C,i}\right)=\sum_{i=1}^{n}\left(A\cdot f_{A,i}f_{C,i}+B\cdot f_{B,i}f_{C,i}+C\cdot f_{C,i}^{2}\right)\;\;$$

If the above equation is written in matrix form, it is as follows:

(16) $$\left(\begin{array}{c} \sum_{i=1}^{n}(y_{i}f_{A,i})\\ \sum_{i=1}^{n}(y_{i}f_{B,i})\\ \sum_{i=1}^{n}(y_{i}f_{C,i}) \end{array}\right)=\;\left(\begin{array}{ccc} \sum_{i=1}^{n}\left(f_{A,i}^{2}\right) & \sum_{i=1}^{n}\left(f_{B,i}f_{A,i}\right) & \sum_{i=1}^{n}\left(f_{C,i}f_{A,i}\right)\\ \sum_{i=1}^{n}\left(f_{A,i}f_{B,i}\right) & \sum_{i=1}^{n}\left(f_{B,i}^{2}\right) & \sum_{i=1}^{n}\left(f_{C,i}f_{B,i}\right)\\ \sum_{i=1}^{n}\left(Af_{A,i}f_{C,i}\right) & \sum_{i=1}^{n}\left(Bf_{B,i}f_{C,i}\right) & \sum_{i=1}^{n}\left(f_{C,i}^{2}\right) \end{array}\right)\cdot \;\left(\begin{array}{c} A\\ B\\ C \end{array}\right)$$

The unknown coefficient of each scintillator can be calculated as follows:

(17) $$\left(\begin{array}{c} A\\ B\\ C \end{array}\right)=\;{\left(\begin{array}{ccc} \sum_{i=1}^{n}\left(f_{A,i}^{2}\right) & \sum_{i=1}^{n}\left(f_{B,i}f_{A,i}\right) & \sum_{i=1}^{n}\left(f_{C,i}f_{A,i}\right)\\ \sum_{i=1}^{n}\left(f_{A,i}f_{B,i}\right) & \sum_{i=1}^{n}\left(f_{B,i}^{2}\right) & \sum_{i=1}^{n}\left(f_{C,i}f_{B,i}\right)\\ \sum_{i=1}^{n}\left(Af_{A,i}f_{C,i}\right) & \sum_{i=1}^{n}\left(Bf_{B,i}f_{C,i}\right) & \sum_{i=1}^{n}\left(f_{C,i}^{2}\right) \end{array}\right)}^{-1}\cdot \;\left(\begin{array}{c} \sum_{i=1}^{n}(y_{i}f_{A,i})\\ \sum_{i=1}^{n}(y_{i}f_{B,i})\\ \sum_{i=1}^{n}(y_{i}f_{C,i}) \end{array}\right)$$

### 3.4. Comparison of Signal-Processing Methods

The pulse shape analysis method is used to effectively distinguish the radiation. Since the decay time of light is different depending on the radiation, the shape of the pulse appears differently depending on the radiation. These are the most used classical methods: the charge comparison method, and the charge-integration method. The FCR–SCR method was developed at the University of Oregon [49] and was used for signal separation of a Phoswich detector composed of three scintillators. In addition, many methods are used as shown in Table 3. It cannot be said which method is the best method, but the appropriate method should be selected according to the type of radiation used and the type of scintillator. In order to determine the degree of separation of the signal, the FOM (Figures of Merit) value is calculated through the equation below [50]:

(18) $$\mathrm{FOM}=\frac{\langle radiation\;1\rangle -\langle radiation\;2\rangle }{\sum_{i}FWHM_{i}}$$

## 4. Phoswich Detectors for Radiological Detection and Measurement

Research is being conducted to minimize the effects of Compton scattering as well as radiation classification using the Phoswich detector, but there are not many research cases using a Phoswich detector. A Phoswich detector consists of two or more scintillators in one optical sensor, and distinguishes radiation using decay time and scintillation efficiency. Each scintillator has a different quantum efficiency(QE) according to the emission wavelength, so signal processing may be difficult. The use of a Phoswich detector can greatly reduce measurement time and cost. Currently, various scintillators are commercialized, but in general, ZnS (Ag) for alpha particles, plastic scintillators for beta particles, and inorganic scintillators for gamma rays are used. γ-ray detection technology has already been developed and commercialized through many studies. Gamma rays are measured by secondary electrons generated by interactions with matter. As a gamma-ray detector, a material with a large atomic number, high density, and a large volume has a high detection efficiency. However, it is not easy to separate β-rays by nuclide because they have a continuous spectrum, unlike gamma rays. β particles are very light and easy to be scattered, and there is a high probability of losing some energy at the detector surface and being lost again by scattering. Since the scattering rate increases as the atomic number of a substance increases, a material with a high atomic number is not suitable as a detector for measuring β-rays. Therefore, when β and γ nuclides are simultaneously measured, they cannot be detected with one detector. Currently, β and γ nuclides are separated and analyzed using different scintillators. In addition, a Phoswich detector enables not only radiation separation measurement, but also background and Compton suppression, and thus related studies are being conducted [51].

**Figure 19:**
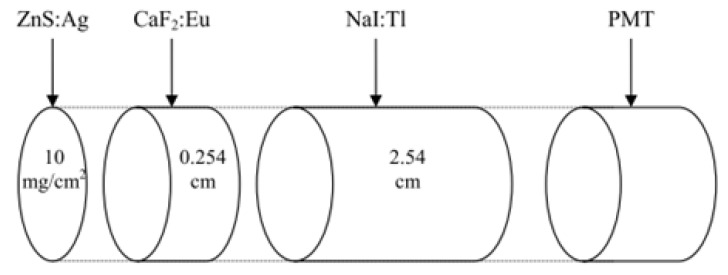
Phoswich detector fabricated by the University of Missouri-Columbia [52].

In 2002, the University of Missouri-Columbia (US) conducted an optimization study for simultaneous alpha/beta/gamma detection using a triple-layer Phoswich detector and MCNP code [52]. As shown in Figure 19, ZnS(Ag) was used for alpha measurement, CaF_2_(Eu) scintillator was used for beta measurement, and NaI(Tl) was used for gamma measurement. Here, by subtracting 26 ± 4% of the total counting value of NaI(Tl) from the counting value of CaF_2_(Eu), the value incorrectly counted by the gamma ray in CaF_2_(Eu) can be corrected. In addition, since the value of the beta reaction incorrectly counted in NaI(Tl) is a reaction due to the bremsstrahlung radiation, the following equation was derived from the CaF_2_(Eu) energy spectrum:

NaI:Tl_ratio_ = 0.055E^4^ − 0.17E^3^ + 0.19E^2^ − 0.059E + 0.008 (19)

Here, *E* is a unit of MeV, and if the NaI(Tl) ratio is multiplied by the total counting rate of CaF_2_(Eu), the beta reaction incorrectly counted in NaI(Tl) can be considered. Based on this study, if the incorrectly counted response for each scintillator is considered in the study on minimizing the effect of Compton scattering using Phoswich detector, the accuracy of the Compton suppression rate will be improved by counting only the desired response for each scintillator.

**Figure 20:**
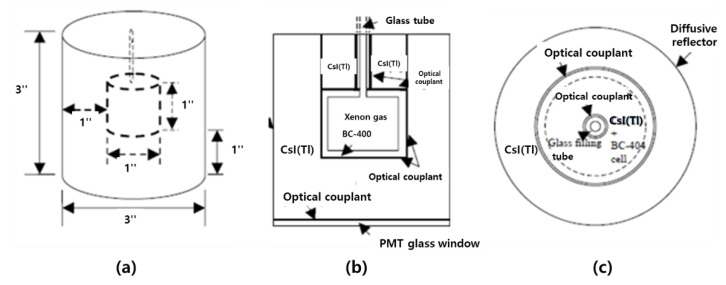
Geometric structure of the ARSA system: (**a**) Detector size; (**b**) Side; (**c**) Top [53].

In 2005, PNNL(US) developed an ARSA (Automated Radioxenon Sampler/Analyzer), a system that monitors xenon radiation in the atmosphere generated by a nuclear test [53]. The system consists of a simultaneous beta and gamma measurement detector to measure four Xe isotopes (^m^Xe131, ^m^Xe-133, Xe-133, Xe-135). In PNNL, a new algorithm was applied to distinguish beta and gamma signals without using the signal rise-time analysis method. By using fast digital readout electronics, the beta single signal, gamma single signal, and beta/gamma simultaneous signal were collected separately. Additionally, the sensitivity and precision of the single-detector performance level were maintained despite the Phoswich structure. Figure 20 shows the geometry of the Phoswich detector. The outer 3” × 3” cylinder is CsI(Tl) crystal, and the inside is 1” × 1” BC-404. In BC-404, xenon gas passes through a thin tube. As a result of conducting simulations using MCNP and experiments with Prototype equipment, it was confirmed that beta particles or converted electrons are absorbed by BC-404 and most of the X-rays or gamma rays are absorbed by CsI. In addition, the simultaneous measurement efficiency of the ARSA system is 82–92%, and the background radiation-removal efficiency is more than 99%. Therefore, the study of PNNL contributed to simplification while maintaining the sensitivity and precision of the existing ARSA.

In 2006, the Korea Atomic Energy Research Institute demonstrated a study on the applicability of the Phoswich detector to directly measure the contamination level inside the pipe. To measure the alpha and beta rays inside the pipe, plastic and ZnS(Ag) were applied to one photomultiplier pipe, and a detection system that distinguished alpha and beta by the PSD method was fabricated and tested. The detection performance of the Phoswich detector for simultaneous alpha/beta-ray measurement according to the location of radioactive contamination was evaluated. In addition, the applicability of the film was evaluated to prevent contamination of the Phoswich detector, and the radiation attenuation for the HDPE film and the aluminized mylar film was confirmed. In the case of alpha rays, when the distance between the plastic scintillator and the source was 2.5 cm, the largest count rate was shown. In the case of the beta ray, the largest detection signal was shown when the source was at a position of 0.5 cm. It was confirmed that the detection of the alpha-ray signal was not affected to 1.5 mg/cm^2^ for the HDPE film for preventing the contamination of the detector and 2.0 mg/cm^2^ for the aluminized mylar film. Additionally, in the case of the beta ray, the attenuation of the beta ray was hardly affected by the thickness of the applied film. Through the results of this study, the applicability of the film for preventing the contamination of the detector by radioactive materials contaminated on the inner surface of the pipe was confirmed. It showed a better alpha/beta signal separation performance than the conventional Phoswich detector. Through this, when combined with equipment that can be transported to a local area, it seems possible to develop a remote device that can secure worker safety and shorten work time [54].

**Figure 21:**
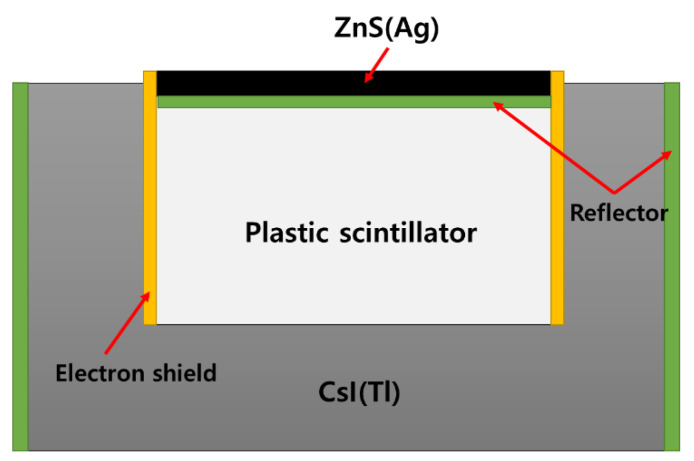
Phoswich detector developed by the National Institute of Radiological Sciences in Japan.

In 2008, the National Institute of Radiological Sciences from Japan developed a detector that combined ZnS(Ag), Plastic, and CsI(Tl) into a single sensor for radiation spectroscopy in Figure 21. Each of the three scintillators has its own decay time (Plastic: 1.8 ns, ZnS(Ag): 200 ns, CsI(Tl): 1000 ns), and separates the pulse according to the fall time using a signal-processing circuit with time resolution. Additionally, the measured radiation was classified into alpha/beta/gamma rays. Measurement experiments were performed using a mixed radiation source of Am-241, Cs-137, and ^90^Sr-^90^Y. As a result of the experiment, there was no significant difference from the measurement efficiency of a general commercial survey meter. Therefore, there is a need for a complementary study [55].

**Figure 22:**
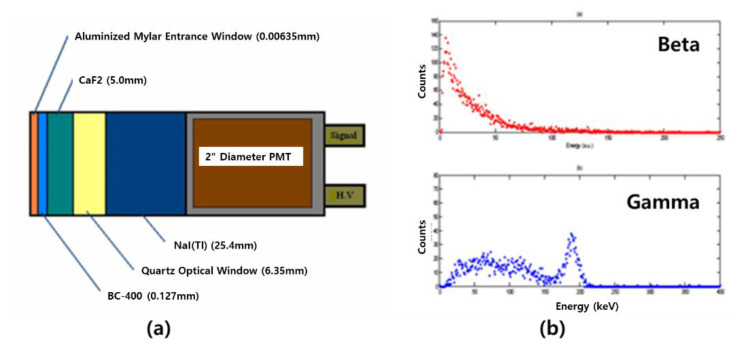
Phoswich detector (**a**) developed by Oregon State University and the filter signal for radiation separation (**b**) [56].

Oregon State University from the US continues to conduct research to detect radioactive xenon generated after nuclear weapons testing using a Phoswich detector, and the major studies conducted at Oregon State University are as follows. In 2006, an ARSA(Automated Radioxenon sampler/analyzer) study was conducted to minimize the overlap between beta and gamma signals using a triple-layer Phoswich detector. CaF_2_ and BC400 scintillators were used for beta measurement in the first and second layers, and NaI (Tl) for gamma measurement was used in the last layer [56]. To minimize the effect of the background, beta and gamma pulses were separated and detected, and a Phoswich detector was developed by connecting a 2-inch PMT with a scintillator. In the case of MCNP coding, the pulses of beta and gamma reacted in each scintillator layer were predicted, and in the case of experiments using pure beta nuclides (^90^Sr/^90^Y) and gamma nuclides (^137^Cs) sources, beta-only signals and gamma-only signals were classified using digital signal-processing technology. Since the decay time of each scintillator is different, it can be classified as a digital signal-processing system, and through this preliminary experiment, it was confirmed that the beta and gamma pulses were separated in Figure 22.

**Figure 23:**
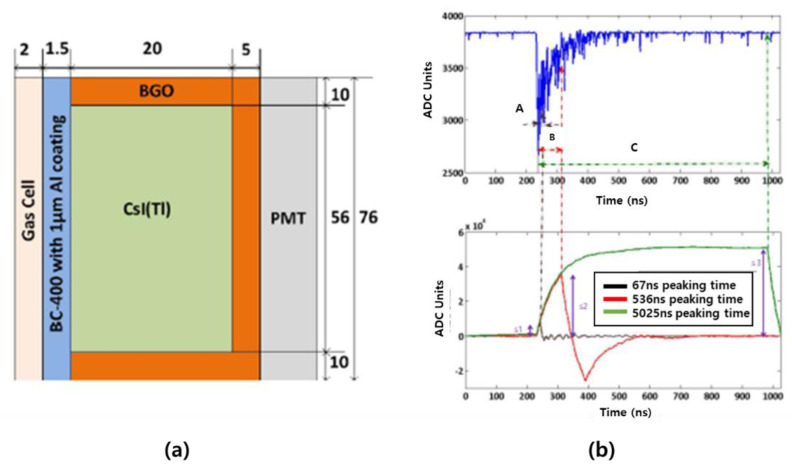
Phoswich detector (**a**) developed by Oregon State University and the filter signal for radiation separation (**b**) [47].

**Figure 24:**
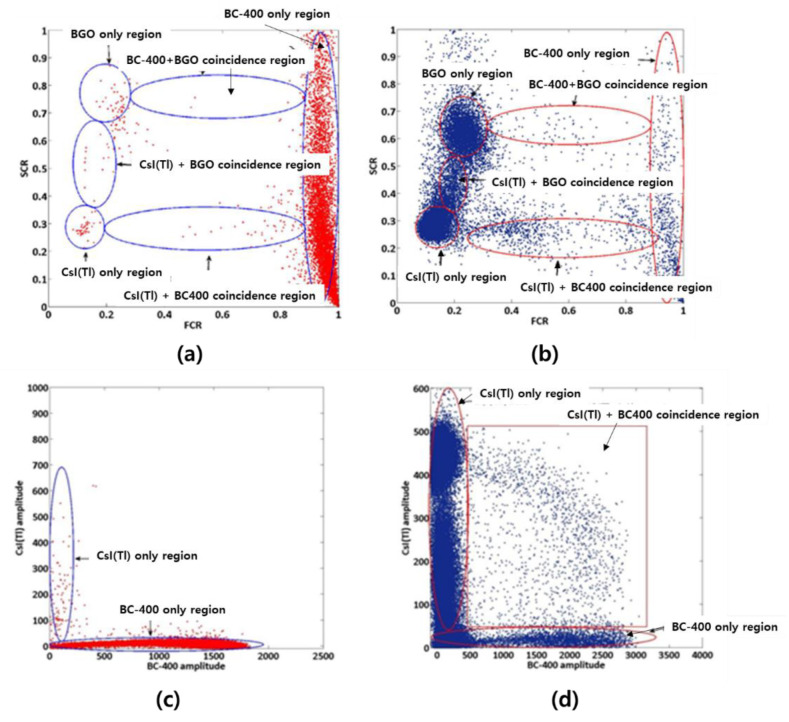
The results calculated by the FCR–SCR method (**a**,**b**) and the least-squares method (**c**,**d**); energy calculation of Cl-36 (**a**,**c**) and Cs-137 (**b**,**d**) sources [47].

In 2013, a Phoswich detector was fabricated with three sensors of BC-400, CsI(Tl), and BGO (Figure 23a), and then a study for Compton suppression was conducted using the PSD method [47]. Oregon State University derives results using two PSD methods (FCR–SCR method, least-squares method) using MATLAB to classify anode output pulses generated from the detector and calculate energy. This method is described in detail in Section 3.2. For Cl-36 (for beta) and Cs-137 (for gamma), the results calculated by the FCR–SCR method and the least-squares method are shown in Figure 24. Single reactions and simultaneous reactions can be classified and observed, and only values for single reactions can be derived by removing the effect on the simultaneous reactions. In the case of a single reaction, most of the reactions are on the axis. The corresponding axis of the layer is filled according to the layer where the interaction occurs. In the case of beta sources, most of the responses were observed on the BC-400 amplitude axis. In the case of the gamma source, most of the responses were observed on the CsI(Tl) amplitude axis. In the case of simultaneous reactions, it appears that most of the reactions will be in the plane instead of the axis. In the FCR–SCR calculation method, an energy measurement technique similar to the pulse identification method or the least-squares method was used. Three (F_BC-400_, F_CsI(Tl)_, F_BGO_) pulse regions were integrated using three digital triangular filters (f1, f2, f3). In addition, the energy emitted from each scintillator was calculated using the appropriate correction factors a and b. For the simultaneous reaction, the energy deposition was calculated differently. For example, in the case of the simultaneous reaction of BC-400 and CsI(Tl) layer, it was calculated with the following equation.

(20) $$E_{\mathrm{CsI}(\mathrm{Tl})}(\mathrm{Energy}\;\mathrm{deposited}\;\mathrm{in}\;\mathrm{the}\;\mathrm{CsI}(\mathrm{Tl})\;\mathrm{layer})=a_{\mathrm{CsI}(\mathrm{Tl})}+b_{\mathrm{CsI}(\mathrm{Tl})}(F_{\mathrm{CsI}(\mathrm{Tl})}-F_{\mathrm{BC}-400})$$

(21) $$E_{\mathrm{BC}-400}(\mathrm{Energy}\;\mathrm{deposited}\;\mathrm{in}\;\mathrm{the}\;\mathrm{BC}-400\;\mathrm{layer})=a_{\mathrm{BC}-400}+b_{\mathrm{BC}-400}(F_{\mathrm{BC}-400}-K_{\mathrm{CsI}(\mathrm{Tl})})$$

Here, a_CsI(Tl)_ and b_CsI(Tl)_ are the calibration factors for the CsI(Tl) layer, and a_BC-400_ and b_BC-400_ are the calibration factors for the BC-400 layer. K is a factor representing the amount contributed to CsI(Tl).

When comparing the FCR–SCR method and the least-squares method, the FCR–SCR method shows better energy resolution. It was analyzed that this is because the single CsI (Tl) or BC-400 response could not be identified in the least-squares method. Finally, as a result of a background suppression experiment using BGO in a 2013 paper from Oregon University, the Compton suppression rate in the 10~400 keV energy range was analyzed as 49 ± 9%. As a result of the Compton suppression experiment using a Cs-137 source, it was analyzed with a Compton suppression rate of 67–30% in the 10–350 keV energy range [48].

In 2013, the CSIC (Spanish National Research Council) from Spain developed a Phoswich detector for high-energy gamma rays and proton identification. Unlike gamma rays, protons interact in matter through continuous deceleration, leaving some of the energy along the track, but accumulating most of the energy in the final absorption process (Bragg Peak). It also prefers to use two layers. Instead of using a very long crystal (25~30 cm), the efficiency was increased by using two short crystals. When choosing the scintillator material to be used in the Phoswich construction, it is important to take into account that the crystals must be optically compatible. That is, the second layer crystal must be transparent to the light emitted from the first layer. In this study, a Phoswich detector was fabricated using LaBr_3_(Ce) and LaCl_3_(Ce), and this scintillator is a scintillator with a very good energy resolution of 3~4% for 662 keV gamma. Since LaCl_3_(Ce) has a light yield of about half compared to LaBr_3_(Ce), the optical peak of the spectrum is located at about half of the number of channels compared to LaBr_3_(Ce). In addition, this author simulates the Phoswich detector composed of LaCl_3_(Ce) and LaBr_3_(Ce) through the Geant 4 code as well as the measurement experiment. The main purpose of the simulation was to adjust the parameters of the simulation for future detector design, and to fully understand the measured data and the physical processes that occur. In this study, it was confirmed that the use of longer crystals resulted in better resolution, but the detection efficiency decreases as the number of nuclear reactions that produce neutron particles increases [57]. 

**Figure 25:**
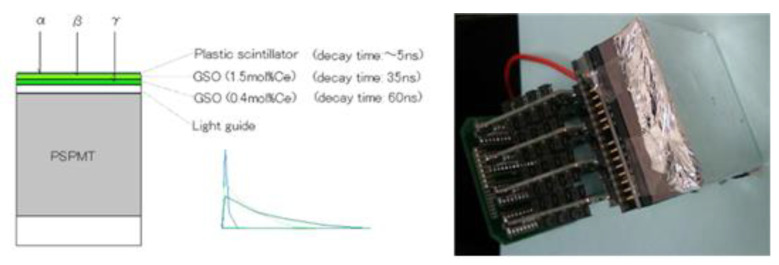
Alpha/beta/gamma imaging detector developed by Nagoya University [58].

In 2015, Nagoya University from Japan developed an alpha/beta/gamma imaging detector using a three-layer Phoswich [Figure 25]. The first layer is a thin plastic scintillator for alpha particle detection; the second layer is a Gd_2_SiO_5_ (GSO) scintillator with 1.5 mol% Ce added for beta-particle detection, and the third layer is a GSO scintillator with 0.4 mol% Ce added for gamma-ray detection. The scintillator was connected to the PMT to produce a detector, and each layer was imaged by classifying alpha/beta/gamma through the PSD method. Gamma rays were detected at 0.6% in the first layer, 60% in the second layer, and 39.4% in the third layer. It was counted that most were the second layer rather than the third layer. This is because the second and third layers are the same as GSO scintillators. As a result of conducting an experiment with the developed Phoswich detector, alpha particles were detected at 6.1% and 0.4% in the second and third layers, respectively. Beta particles were detected at 0.4% and 11.9% in the first layer and third layer. Gamma rays were detected at 0.6% in the first layer. The reason beta showed the greatest response (11.9%) in the third layer is that the second layer was 0.5 mm thick. It was too thin to absorb all of the high-energy beta particles emitted from ^90^Y (2.8 MeV). Later, it is necessary to further study the results when placing the thick GSO in the second layer [58].

**Figure 26:**
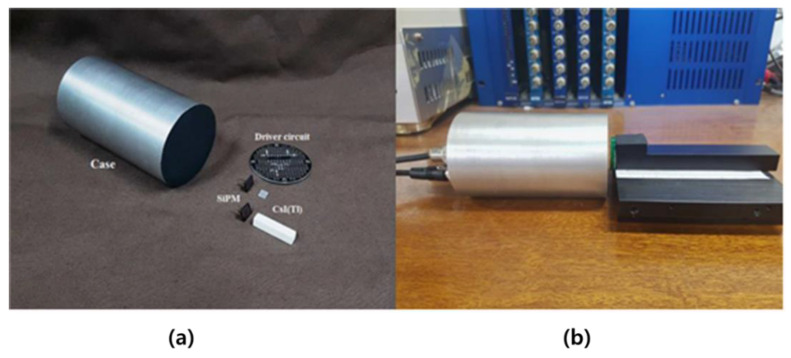
Beta/gamma detector connected with CsI(Tl), SiPM sensor (**a**); driving circuit (**b**) [59].

In 2018, Myongji University tried to use CaF_2_ (Eu), which is more efficient than YAP (Ce) and YAG (Ce), as the beta-ray-detection scintillator, and CsI(Tl) as the gamma-ray-detection scintillator. Figure 26 shows a picture of the beta/gamma detector configuration using SiPM. However, due to the different light yields of the two scintillators, the energy calibration of Cs-137 and Sr-90 was not possible, so CsI(Tl) was produced with a thickness of 30 mm (for gamma) and 1 mm (for beta), and a beta/gamma double detector was fabricated. In addition, it was possible to compare the beta spectrum and the gamma spectrum by attaching SiPM to the manufactured beta/gamma dual detector, but the detection of the simultaneous measurement signal was unsuccessful [59].

**Figure 27:**
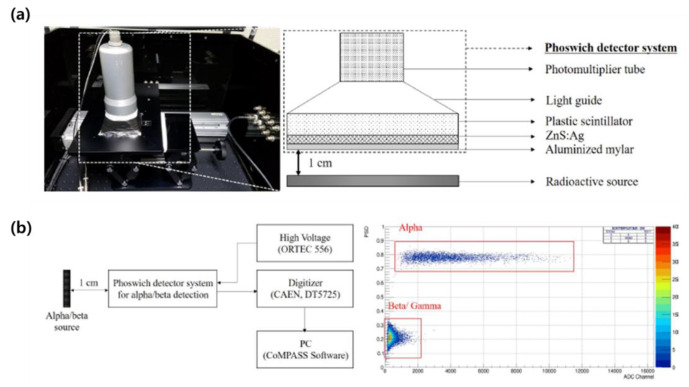
Description of the Phoswich detector system for simultaneous alpha/beta detection (**a**) and a schematic diagram of alpha and beta counting setup and PSD results (**b**) [60].

In 2019, KAIST from the Republic of Korea optimized the thickness of the scintillator by manufacturing a Phoswich-type detector that can simultaneously measure alpha and beta radioactive contamination in order to develop a technology that can quickly and efficiently measure the radioactive contamination of a large area. As the scintillator of the Phoswich detector, ZnS:Ag(EJ-600) for alpha, a plastic scintillator (EJ-212) for beta was selected in consideration of detection efficiency and decay time [Figure 27]. Pulse shape discrimination is a technique for distinguishing among different types of radiation by using the fluorescence decay-time difference of scintillators. Alpha particles have a relatively short range compared to beta particles. In this case, alpha particles transfer their energy to the ZnS:Ag layer with a decay time of 200 ns, and beta particles pass through the ZnS:Ag layer and deposit their energy in the plastic scintillator with a decay time of 2.4 ns. By using the luminescence decay time difference, PSD was performed in the digitizer (14 bit, 250 MS/s waveform digitizer). Alpha-to-beta crosstalk was less than 5%, and beta-to-alpha crosstalk was less than 2%. In this study, static MDC was evaluated by equation (22) for each scintillator size and radiation source, and the results are shown in Table 4 [60].

(22) $$MDC_{static}\frac{3+4.65\sqrt{C_{B}}}{\epsilon_{i}\epsilon_{s}\left(\frac{A}{100cm^{2}}\right)T}$$

Here, *C_B_* is the background count in time, *ε_s_* is the surface efficiency, *A* is the detector probe area in cm^2^, and *T* is the counting time in minutes.

When the scintillator size was increased from 15 × 15 cm to 20 × 20 cm, the static MDC of beta-emitting radionuclides remained similar, but the static MDC of alpha radionuclides benefited from the increase in scintillator size. Static MDC sizes of 15 × 15 cm and 20 × 20 cm were not significantly different, but the larger the size, the lower the spatial resolution was evaluated. If the Phoswich detector developed in this study is manufactured in an array form for large area measurement, it is expected to be useful in the field of decommissioning nuclear facilities [60].

If the Phoswich detector is manufactured in the form of an array, it is expected that it will be useful for establishing a decommissioning plan, classifying radioactive waste, and restoring the site based on the result of radioactive contamination distribution measurement.

**Figure 28:**
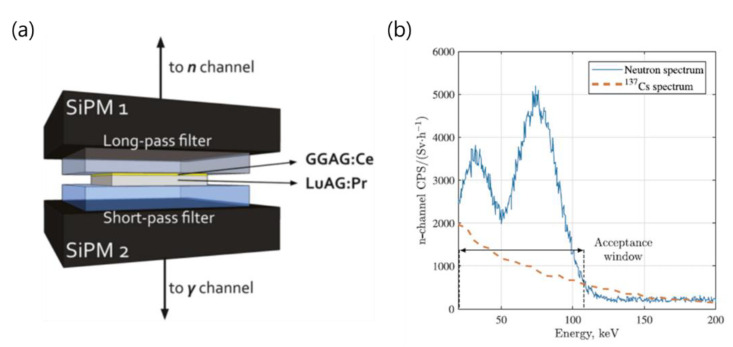
Schematic of the Phoswich arrangement (**a**) and comparison between the n-channel multichannel spectra obtained by irradiating with neutrons (solid blue line) and 137Cs (dashed red line) without implementing the anti-coincidence logic (**b**) [61].

In 2019, Polytechnic University from Italy proposed a new approach for particle identification in mixed-radiation fields [61]. A thin single crystal scintillator of Lu_3_Al_5_O_12_:Pr(LuAG:Pr) and Gd_3_Al_2_Ga_3_O_12_:Ce (GGAG:Ce) was used. Both crystals are sensitive to gamma rays, but because neutrons interact only with GGAG:Ce due to the presence of Gd, which has a large reaction cross-section with neutrons, it is possible to distinguish between incident neutrons and gamma rays. Therefore, optical filtration and an anti-coincidence algorithm are therefore used to perform particle discrimination, rejecting coincidence signals arising from gamma rays, which simultaneously deposit energy in both crystals, and counting anti-coincidence signals due to neutrons, which deposit energy only in the GGAG:Ce. In addition, since the scintillator is thin, the background gamma rays incident on the two scintillators at the same time are removed as a coincidence signal to minimize the effect of the background. The detection system combines two scintillators (GGAG:Ce of 5 × 5 × 0.1 mm^3^ and LuAG:Pr of 5 × 5 × 0.5 mm^3^) with two separate SiPMs through an optical filter, as shown in Figure 28a.

In this study, the lower acceptance window boundary was chosen to remove the noise of the SiPM, and the upper limit was chosen to replace the main IC electron emission peak except for the high-energy Compton electrons in the gamma background. Figure 28b compares the multichannel spectra obtained by examining neutrons and ^137^Cs in the case where no anti-coincidence logic is implemented. The partial Compton edges produced by the ^137^Cs mostly overlap the acceptance window. The low crystal thickness prevents the formation of complete Compton edges and photo peaks. This study has proven through simple cases that particle identification can be pursued with an emission-based approach.

Internal conversion (IC) electrons are generated due to interaction with neutrons and one or more photons are generated as the empty space of electrons is filled by other electrons. The signals of these reactions are recorded as neutron signals. In other words, it is a system to reduce the influence of photons generated by interaction with neutrons. The main point of this study is that electronic devices can be simplified. This is because algorithms and additional elements/electronics for pulse shape analysis are not required. Only the window that receives the signal was adjusted to distinguish the signal [61].

**Figure 29:**
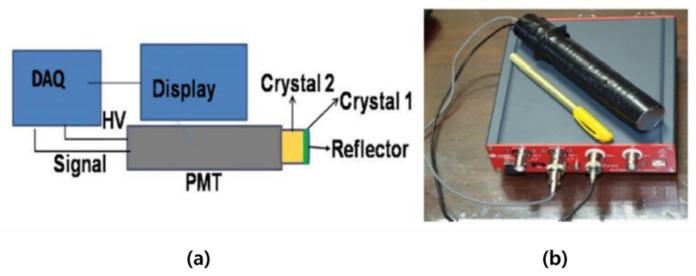
(**a**)The scheme and (**b**) actual setup of the Phoswich detector [62].

**Figure 30:**
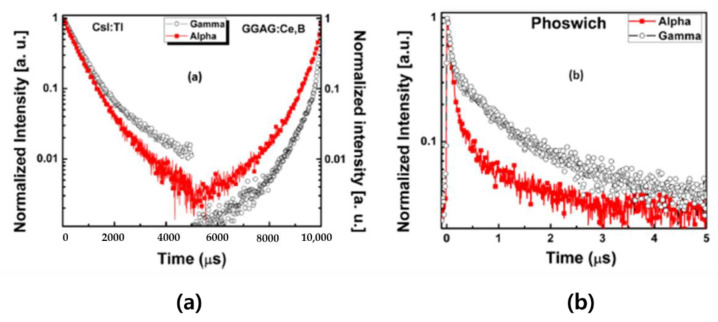
Scintillation decay curves measured with alpha and gamma irradiations on the CsI:Tl and the GGAG single crystal (**a**), and the GGAG/CsI:Tl Phoswich detector (**b**) [62].

In 2020, the BARC (Bhabha Atomic Research Centre) from India developed a Phoswich detector to discriminate between alpha and gamma. The Phoswich detector was fabricated in a new combination by combining GGAG (Gd_3_Ga_3_A_l2_O_12_:Ce) with CsI:Tl. The detector was optically coupled to a single photomultiplier tube and desktop digitizer for a compact configuration. The gamma interacting in the front GGAG crystal and the back CsI crystal was identified as a distinct FOM that can lead to significant improvements in spatial resolution in medical imaging applications. Figure 29 is a schematic and actual photograph of the Phoswich detector design. GAGG and CsI:Tl have different pulse properties, which show their excellent ability to discriminate between alpha, beta, gamma, and neutrons individually. The emission of light from the front GGAG crystal peaking at about 550 nm is in the transmission region of the CsI crystal bonded to the PMT. Therefore, the two scintillators are a good combination. In this study, radiation identification was classified through the PSD method. As a result, as shown in Figure 30a, the difference in scintillation decay time measured by alpha and gamma rays incident on CsI:Tl and GGAG, respectively, are shown. In the case of CsI:Tl, the alpha particle consequently produces a higher ionization excitation density that affects the decay kinetics of relaxation. This high ionization density generally suppresses emission and makes the decay time faster than gamma rays. However, the effect was observed to be opposite in the GGAG crystal, which lowers scintillation decay by comparing alpha and gamma, as shown in Figure 30b. The dependence of the decay time on alpha and gamma radiation results in a large difference in the decay time, which greatly improves the ability of Phoswich to discriminate [62].

In most studies on gamma ray measurement, an inorganic scintillator is used, and an organic scintillator is used for beta-ray measurement. In addition, multiple detectors are used to minimize the background and Compton effect caused by the interaction between radiation and the scintillator. In most studies on the Phoswich detector, various radiation-identification methodologies have been developed for use in a mixed-radiation field. In the radiation-identification studies, the PSD (pulse shape discrimination) method was used or modified, and the energy deposited by each scintillator was also analyzed by the signal-processing method. Integrated multiple detection systems and Phoswich detectors in radiological measurements are summarized and the studies presented above are shown in Table 5 and Table 6.

## 5. Conclusions and Outlook

In this review, we summarize integrated multiple detection systems and Phoswich detectors in radiological measurements for their device performance. Additionally, we further provide the integration of Phoswich detectors with readout-integrated circuits and the associated performance for radiological detection and measurement. A radiological-sensing application using Phoswich detectors to improve the background reduction performance of the existing radiation detection system and to compensate for the shortcomings was investigated. A Phoswich detector is a type of detector that connects two or more scintillators with one optical sensor. In the research case investigated above, it is mainly the development of a system that detects high-energy beta or gamma nuclides. Currently, it has been investigated that scintillators such as ZnS(Ag), plastic, NaI(Tl), CsI(Tl), CaF_2_(Eu), LaBr_2_(Ce) are being used in Phoswich-detector-development research. Research on technology development for separating alpha, beta, and gamma according to the size and composition of each scintillator is being conducted worldwide. However, when two or more scintillators are connected in series, since radiation is measured by crosstalk, the influence on radioactive crosstalk must be minimized. Here, crosstalk refers to a phenomenon in which a signal received from one optical sensor has an unintended effect in another channel (a region for an unwanted scintillator). Currently, background reduction studies using HPGe detectors and guard detectors are being conducted more actively than using Phoswich detectors. Phoswich detection systems are generally used to classify radiation (alpha, beta, and gamma), but if the structure and function of the Phoswich detector are applied to the Compton suppression system, it is possible to develop a simpler system with similar performance to the existing technology. Although studies on measurement and analysis methods using Phoswich detectors are being carried out, it is considered that additional studies are needed to derive reliable results. In particular, it is necessary to derive results verification methods and algorithms that can minimize the effect on radiation crosstalk. Moreover, it is necessary to develop a background reduction study using a Phoswich detector and a signal-processing method, and a study on the development of simultaneous alpha/beta/gamma measurement technology. Importantly, the implementation of digital processing working in an integrated multiple-detection system for radiological measurement brought significant advantages to analyzing data from Phoswich detectors. Thus, digitization will be one of the main works which can effectively perform accurate quantitative measurements in radiological-sensing applications. Data acquisition, integration like the processing and joining of data sets, and data analysis using algorithms are made easier and less time-consuming by processing only one Phoswich detector. Eventually, digitalization in this field of radiation detection and measurement can make faster and easier the digital acquisition and processing unit with Phoswich detectors in sensing applications. In the end, we expect this review will pave the way to understanding the recent status and future challenges for Phoswich detectors for radiological detection and measurement.

## Figures and Tables

**Table 1:** Types and characteristics of inorganic scintillators [14,15,16,17,18,19,20,21,22,23,24,25].

Material	Peak Emission Wavelength (nm)	Decay Time (ns)	Density (g/cm^3^)	Light Yield (photon/MeV)	FWHM at 662 keV (%)	Index of Refraction
NaI(Tl)	415	230	3.67	38,000	7.0	1.85
BGO	480	300	7.13	8200	9.5	2.15
CsI(Tl)	540	800	4.51	60,000	9.0	1.8
LaBr_3_(Ce)	358	35	5.3	61,000	4.0	-
BaF_2_	310	630	4.9	10,000	7.7	1.49
CdWo_4_	530	15,000	7.9	7000	-	-
Gd_2_SiO_5_(Ce)	440	60	6.7	10,000	8.5	-

**Table 2:** Comparative table of three types of optical sensors [43].

	PMT	APD	SiPM
Gain	10^6^	50~1000	~10^6^
Rise time (ns)	~1	~5	~1
Quantum efficiency (% at 420 nm)	~25	~70	~25~75 (photon detection efficiency, PDE)
Bias (V)	>1000	300~1000	30~80
Temperature sensitivity (%/°C)	<1	~3	1~8
Magnetic field sensitivity	yes	no	no
Sensitive area	cm^2^	mm^2^	mm^2^
Price/channel ($)	>200	~100	~50

**Table 3:** PSD analysis methods in the time and frequency domains.

Domain	Contents	Method
PSD analysis methods applied in the time domain	Zero crossing discrimination technique/constant fraction discriminators	Conventional
	Rise time discrimination	
	Charge comparison method (CCM)	
	Constant-fraction timing discriminator	
	Pulse gradient analysis (PGA)	Digital
	Artificial neural network (circuit of neurons)	
	Fuzzy logic (FL)	
	Curve fitting technique	
	Pattern recognition technique	
PSD analysis methods applied in the frequency and time domains	Frequency gradient analysis (FGA)	Digital
	Principal component analysis (PCA)	
	Cross-correlation anaysis (CCA)	
	Wavelet-based analysis (continuous and discrete)	

**Table 4:** Static MDCs for 1 min measurements depending on probe size [60].

Scintillator Size (cm)	Static MDC (dpm/100 cm^2^)			
	^241^Am (alpha)	^90^Sr/Y (beta)	^99^Tc (beta)	^14^C (beta)
10 × 10	76 ± 73	445 ± 113	1584 ± 404	13,912 ± 3611
15 × 15	43 ± 32	248 ± 60	1304 ± 319	7836 ± 1938
20 × 20	33 ± 32	252 ± 60	1324 ± 317	8060 ± 2115

**Table 5:** Summary of integrated multiple-detection systems in radiological measurements.

Scintillator or Phoswich Material	Radiation	Algorithm	Main Results	Ref.
Plastic Scintillator (NE102A)	β	- In order to utilize the principle similar to that of the liquid scintillator, a 3 cm × 2 cm × 1 cm sized sample hole was made in the center of the plastic scintillator to enable omnidirectional measurement	- MDA value of 1.21 Bq/cm^2^ is obtained - Verification of the performance of the beta detector through MCNP simulation	[29]
Gd_2_O_2_S(Tb), Y_3_Al_5_O_12_(Ce), CsI(Tl)	β	- A long-distance measurement test using optical fiber was performed - Use of the anti-coincidence method	- Selecting the optimal scintillator by comparing the H-3 measurement results for each inorganic scintillator - Derive the dose equation according to the distance between the detector and source	[32]
Plastic scintillator (Polystyrene-based)	β	- Minimization of noise effect by using simultaneous counting circuit - Plastic sheet system to be used as an alternative to LSC	- MDA value of 0.0116 Bq/mL is obtained for the 2 mL sample	[31]
Plastic scintillator (Epoxy-based)	β	-The focus is on plastic production -The detection efficiency was calculated using the Sr-90 source -Performance evaluation of plastic scintillator by thickness of plastic scintillator and content of scintillation material was performed	- Optimum scintillation material concentration is (polymer: PPO: POPOP = 0.79 wt%: 0.2 wt%: 0.01 wt%) - Optimum thickness is 4 mm	[33]
CaF2(Eu) detector	β	- Focusing on tests to derive optical sensors suitable for CaF_2_ (Eu)	- The MDA of SiPM-CaF_2_ (Eu) was 1319 Bq/L, and the MDA of PMT-CaF_2_ (Eu) was 330 Bq/L - It was analyzed that PMT-CaF_2_(Eu) is a more suitable system for low-concentration Sr-90 measurement than SiPM-CaF_2_(Eu) - Efficiency increased by 30% through the removal of Compton at 964 keV - When low background radiation is required, a product using NaI(Tl) as a guard detector is recommended	[34,35]
HPGe, BGO	γ	- Use of anti-coincidence method - Guard detectors to reduce background effects are used		[36]
HPGe, NaI(Tl)	γ	- Use of anti-coincidence method - Guard detectors to reduce background effects are used		[37]
HPGe, NaI(Tl)	γ	- Use of anti-coincidence method - RF (Reduction Factor)	- MDA decreased from 8.2 ± 0.4 Bq/kg to 1.8 ± 0.2 Bq/kg	[38]
HPGe, NaI(Tl)	γ	- The peak of the Compton ratio was calculated in each energy domain using the Compton suppression method and the non-Compton suppression method	- The peak of the Compton ratio in the energy region of cesium nuclides was 0.26 (604.72 keV), 0.20 (795.86 keV), and 0.06 (569.33 keV) - The MDA of Cs-137 was calculated as 1.5 μBq/m^3^ (Compton non-suppression) and 0.7 μBq/m^3^ (Compton suppression), respectively	[39]

**Table 6:** Summary of Phoswich detectors in radiological measurements.

Scintillator or Phoswich Material	Radia-tion	Algorithm	Main Results	Ref.
ZnS(Ag), CaF_2_(Eu), NaI(Tl)	α, β, γ	Correction factor (correction of gamma effect incorrectly counted in the beta detector)	- Analysis that about 26±4% gamma reaction occurred in the beta detector(CaF_2_(Eu)) - Derivation of a correction factor methodology that removes the gamma response incorrectly counted in the beta detector from the gamma detector(NaI(Tl))	[52]
BC-404, CsI(Tl)	β, γ	Identification based on signal rise time	- Optimization of detection-sensor geometry for xenon detection (β, γ) - Use of signal rise-time analysis method to distinguish whether the signal responding to the sensor is an individual signal or simultaneous signal - Comparison of detection efficiency through MCNP simulation (82~92% match)	[53]
BC-400, CsI(Tl), BGO	β, γ	- FCR (Fast component ratio) and SCR (Slow component ratio) - Least-squares method	- Detector configuration with well-type Phoswich structure for radioactive Xenon detection - All signals are processed with a self-developed FPGA device to classify beta/gamma signals using filters with different peaking times - Shows MDC of less than 1 mBq/m^3^ for radioactive Xenon isotopes	[47]
Plastic, GSO (1.5 mol% Ce), GSO (0.4 mol% Ce)	α, β, γ	- Identification based on signal decay time - Alpha/beta/gamma detection using triple-layer Phoswich structure - Calculate the response rate for each layer of alpha/beta/gamma	- Gamma rays were detected at 0.6% in the first layer, 60% in the second layer, and 39.4% in the third layer - Alpha particles were detected at 6.1% and 0.4% in the second and third layers, respectively. Beta particles were detected at 0.4% and 11.9% in the first layer and third layer. Gamma rays were detected at 0.6% in the first layer	[54]
ZnS(Ag), Plastic scintillator	α, β	- Pulse shape discrimination is a technique for distinguishing among different types of radiation by using the fluorescence decay-time difference of scintillators	- Decay time of ZnS(Ag) is 200 ns and the decay time of plastic scintillator is 2.4 ns - Alpha-to-beta crosstalk was less than 5%, and beta-to-alpha crosstalk was less than 2%. - It was analyzed that the spatial resolution decreases as the scintillator size increases	[60]
LuAG(Pr), GGAG(Ce)	n, γ	- Setting the acceptance window of MCA for signal-filter and noise minimization - The two scintillators used are sensitive to gamma rays, but the neutron reacts only to GGAG containing Gd, so the neutron removes the gamma signal measured simultaneously from the GGAG and counts only the neutron signal	- The main point of this study is that electronic devices can be simplified. This is because algorithms and additional elements/electronics for pulse shape analysis are not required. Only the window that receives the signal was adjusted to distinguish the signal - A Phoswich set-up for neutron/gamma discrimination by the introduction of a three-slice Phoswich, i.e., LuAG:Pr-GGAG:Ce-LuAG:Pr including a logic block diagram of the readout circuit	[61]

## Data Availability

Not applicable.
